# Insights into the osteoblast precursor differentiation towards mature osteoblasts induced by continuous BMP-2 signaling

**DOI:** 10.1242/bio.20134986

**Published:** 2013-07-03

**Authors:** Omar F. Zouani, Lila Rami, Yifeng Lei, Marie-Christine Durrieu

**Affiliations:** 1 Bioingénierie Tissulaire (BioTis), INSERM U1026, Université de Bordeaux, 146 rue Léo Saignat, 33076 Bordeaux, France; 2 Institut Européen de Chimie et Biologie (IECB), CNRS, UMR 5248, Université de Bordeaux I, 2 rue Robert Escarpit, 33607 Pessac, France

**Keywords:** Mature osteoblasts, BMP-2, Runx2, Actin cytoskeleton, Super-resolution optical profilometry

## Abstract

Mature osteoblasts are the cells responsible for bone formation and are derived from precursor osteoblasts. However, the mechanisms that control this differentiation are poorly understood. In fact, unlike the majority of organs in the body, which are composed of “soft” tissue from which cells can easily be isolated and studied, the “hard” mineralized tissue of bone has made it difficult to study the function of bone cells. Here, we established an *in vitro* model that mimics this differentiation under physiological conditions. We obtained mature osteoblasts and characterized them on the basis of the following parameters: the strong expression of osteoblastic markers, such as Runx2 and Col-I; the achievement of specific dimensions (the cell volume increases 26-fold compared to the osteoblast precursors); and the production of an abundant extracellular matrix also called osteoid. We demonstrated that the differentiation of osteoblast precursors into mature osteoblasts requires the continuous activation of Bone Morphogenetic Protein (BMP) receptors, which we established with the immobilization of a BMP-2_mimetic peptide_ on a synthetic matrix mimicking *in vivo* microenvironment. Importantly, we demonstrated that the organization of the F-actin network and acetylated microtubules of the cells were modified during the differentiation process. We showed that the perturbation of the F-actin cytoskeleton organization abolished the differentiation process. In addition, we demonstrated that expression of the Runx2 gene is required for this differentiation. These findings demonstrate the retro-regulation of cytoplasmic and genic components due to the continuous induction of BMP-2 and also provide more detailed insights into the correct signaling of BMPs for cell differentiation in bone tissue.

## Introduction

Unlike the majority of organs in the body, which are composed of “soft” tissue from which cells can easily be isolated and studied, the “hard” mineralized tissue of bone has made it difficult to study the function of bone cells. Moreover, we have the lack of tools to manipulate these bone cells (Bonewald, 2011; Lutolf and Blau, 2009). Furthermore, the complexity of the spatial arrangement and the composition of the microenvironment of the bone cells add to the challenge. Several strategies have been developed for the design of *in vitro* culture systems to mimic the extracellular microenvironment of bone cells (Lutolf and Blau, 2009). In bone tissue, the extracellular matrix (ECM) is composed of proteins that promote cell adhesion, and growth factors such as BMPs that promote cell differentiation (Chen et al., 2004; Wagner et al., 2010). These various proteins exhibit different distributions (Marie, 2009). In addition to the extracellular antagonists controlling the binding of the ligand to receptor complexes to regulate BMP signaling (Sengle et al., 2008), different adhesion components of ECM modulate BMP signaling (Wang et al., 2008; Hynes, 2009). Therefore, the retention of BMPs by antagonists or ECM adhesion proteins may be an important mechanism for controlling receptor availability at the cell surface and inducing the attenuation of signal transduction, thereby delaying the internalization of the BMP–receptor complex. Thus is the “solid induction mode” (Crouzier et al., 2011; Zouani et al., 2013a). The concept of presenting a growth factor “in the solid induction mode,” which we refer to as “matrix-bound,” has been proposed in pioneering work performed by Kuhl et al. on tethered epidermal growth factor (EGF); however, this work has been neglected for many years (Kuhl and Griffith-Cima, 1996). This matrix-bound mode of growth factor presentation is closer to physiological conditions because most growth factors in the ECM are bound to proteins and glycosaminoglycans. Recent experimental studies have shown that the matrix-bound form of presentation improves the efficacy of different growth factors when compared to the soluble form and that their biological function is enhanced (Chen et al., 2010; Davis et al., 2006; Fan et al., 2007). The “punctual induction mode” is the most commonly studied and best known method for the presentation of BMPs in the soluble state (Shah et al., 1999; Sieber et al., 2009; Wagner et al., 2010) (supplementary material Fig. S1A). In bone tissue, because of the presence of high levels of collagens containing BMPs interaction specific site, these BMPs are sequestered in the ECM, and the solid induction mode is favored over the punctual induction mode. Diseases in different tissues are caused by malfunctions in the BMP signaling pathways (Ramirez and Rifkin, 2009; Maciel et al., 2010; Mazzaferro et al., 2010; Sethi and Kang, 2011).

BMPs are secretory signaling molecules belonging to the TGF-β superfamily of growth factors. BMPs play important roles in the induction of bone formation (Wagner et al., 2010). These growth factors have the potential to induce different bone cell differentiation processes at different stages (Carragee et al., 2011). BMPs bind to dimeric receptor complexes composed of type I and type II transmembrane serine/threonine kinase receptors (Wagner et al., 2010; Sieber et al., 2009). The receptors form homomeric and heteromeric complexes in distinct membrane areas and are differentially modulated by their ligands. BMP-2 binds to preformed heterocomplexes of the type I and type II receptors, initiating Smad-dependent signaling. The Smad pathway is initiated by the phosphorylation of regulatory Smad1/5/8, which associate with the common mediator Smad (Smad4), translocate into the nucleus, and regulate the transcription of specific BMP target genes, such as Runx2, by recruiting additional activators and repressors (Sieber et al., 2009; Komori, 2010).

In the osteoblast lineage, cells progress through various stages of differentiation and maturation. Osteoblast progenitors are derived from adult mesenchymal stem cells, followed by osteoblast precursors, mature osteoblasts and osteocytes (Kato et al., 2001). *In vivo*, mature osteoblasts are distinguished by their dimensions in the bone (Gohel et al., 1995; Maes et al., 2010) and are found on the ECM (referred to as osteoid), which is produced by the osteoblasts themselves. BMPs (particularly BMP-2, which is more abundant in the ECM of bone tissue) play an important role during these differentiation stages and are particularly important for the generation of mature osteoblasts *in vivo* (Sieber et al., 2009) (supplementary material Fig. S1B,C). However, the mechanisms that control this differentiation (osteoblast precursors into mature osteoblasts) are poorly understood. Recently, the solid induction mode was suggested for BMPs to induce the correct intracellular signals promoting precursor osteoblasts differentiation into mature osteoblasts (Zouani et al., 2010). In the present study, we used a mature osteoblast differentiation model using a modified polymer that mimics the osteo-induced ECM and that is based on the solid BMP-2 induction mode. With this approach, the polymer polyethylene terephthalate (PET) was functionalized using a BMP-2_mimetic peptide_ favoring osteogenesis. Using this system, the activation of BMPr-1A is continuous, mimicking the physiological ECM-containing BMP-2 that surrounds the osteoblast precursors, because of the arrest of the BMP-2/BMP receptor complex process. In addition, with the generation of mature osteoblasts, the regulation of the osteoblast precursor differentiation can be characterized, giving an indication at the physiological level.

Using this differentiation model, we showed that the mature osteoblasts generated *in vitro* are characterized by cell swelling. This study of cell size, providing a three-dimensional view, was performed using an optimized optical profilometry (Schmidt and Black, 1992; Chen et al., 2009; Xiao et al., 2010; Zouani et al., 2012; Pan et al., 2011; Lei et al., 2013) and a specific cell treatment protocol, which allowed for a vertical nanometer resolution. Other features that are characteristic of a mature osteoblast include (i) an increase in Runx2 and Col-I expression and (ii) an increase in ECM (osteoid-like matrix) production. These features signify a transformation from precursor to mature osteoblasts. Next, we were interested in characterizing the relationship between the genes that characterize the differentiation of the cells and the increased volume of the mature osteoblasts. We investigated the major components of the cytoskeleton, such as F-actin organization and the presence of acetylated tubulin. Therefore, we showed that whereas osteoblast differentiation is regulated by Runx2 gene, it is also dependent on cytoskeletal components that are responsible for the size and shape of the cells during their transformation. In summary, this study demonstrates that the continuous induction mode of BMP-2 (the correct BMP-2 signaling mode in bone tissue) is essential for the generation of mature osteoblasts. Our findings suggest that this phenomenon is likely common to all BMPs signaling that are regulated by the ECM on bone, thereby regulating a number of bone–cell differentiation processes.

## Results

### Generating mature osteoblasts

**Fig. 1.**
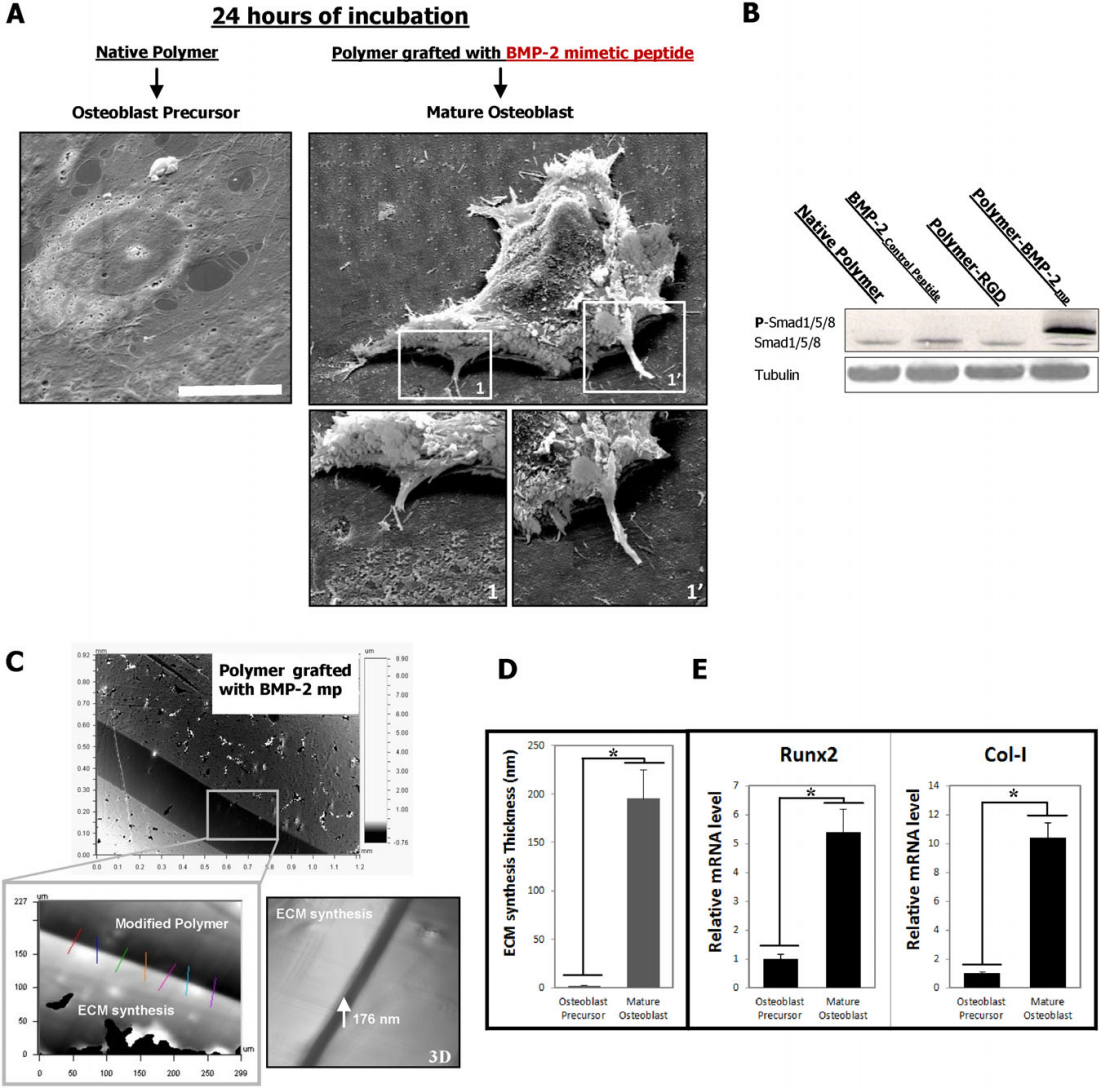
The generation of mature osteoblasts. (**A**) SEM micrographs showing cultured osteoblast precursors on different modified polymer surfaces and their transformation. Scale bar: 20 µm. (**B**) Smad1/5/8 pathway has been activated after 24 hours for osteoblast precursors cultured on polymer grafted with BMP-2_mimetic peptide_ as seen by phospho-Smad1/5/8 blotting. (**C**) An example of an OPS micrograph showing the ECM formed by the cells after 24 h culture on the polymer surface grafted with the BMP-2_mimetic peptide_. The yellow square represents a zoom of a particular region. 7 to 10 measurements were performed in order to determine the thickness of the newly synthesized ECM. Down, right, a 3D reconstruction image of the region in the yellow square. (**D**) A graph quantifying the evolution of the ECM thickness at 24 h after cell seeding. Note that the ECM protein aggregates are scarce on the native PET and are abundant on the polymer surfaces grafted with RGD and/or the BMP-2_mimetic peptide_ (*P<0.001). (**E**) The quantitative PCR analysis for Runx2 and Col-I; significant differences were observed between the native polymer (control) and the polymer grafted with the BMP-2_mimetic peptide_ alone (*P*<0.005).

The interaction between the ECM components and the inducers (e.g., BMP-2) is essential for correct signaling. Here, a synthetic ECM-BMP-2 analogs in two-dimensional (2D) culture systems controlling cell fate was created and their effects have been evaluated on the behavior of a mouse osteoblastic precursor cell line (MC3T3-E1). After culturing for 24 h, the osteoblast precursors were seeded onto the BMP-2_mimetic peptides_ functionalized polymer and transformed into mature osteoblasts. The cells exhibited a radically changed phenotype with an increased morphology, volume and height (Fig. 1A). In our model, the activated signaling pathway is the Smad1/5/8 (Fig. 1B). In addition, the ECM quantity synthesized (the osteoid-like matrix) by the cells was studied on this novel cell type. The differentiated osteoblast precursors, characterized by their increased height, produced intensive amounts of ECM compared to the osteoblast precursors cultured on a native polymer (on plastic or on a modified polymer grafted only with RGD peptides derived from natural ECM proteins; data not shown) (Fig. 1C,D). Finally, the osteoblast precursors transformation into mature osteoblasts was verified by analyzing the expression of the osteogenic biomarker core binding factor α1 (Cbfa-1), also called Runx2, as well as the expression of Col-I. We observed an increase in Runx2 and Col-I gene expression after 24 h in culture (Fig. 1E).

### Mature osteoblasts are characterized by cell swelling

**Fig. 2.**
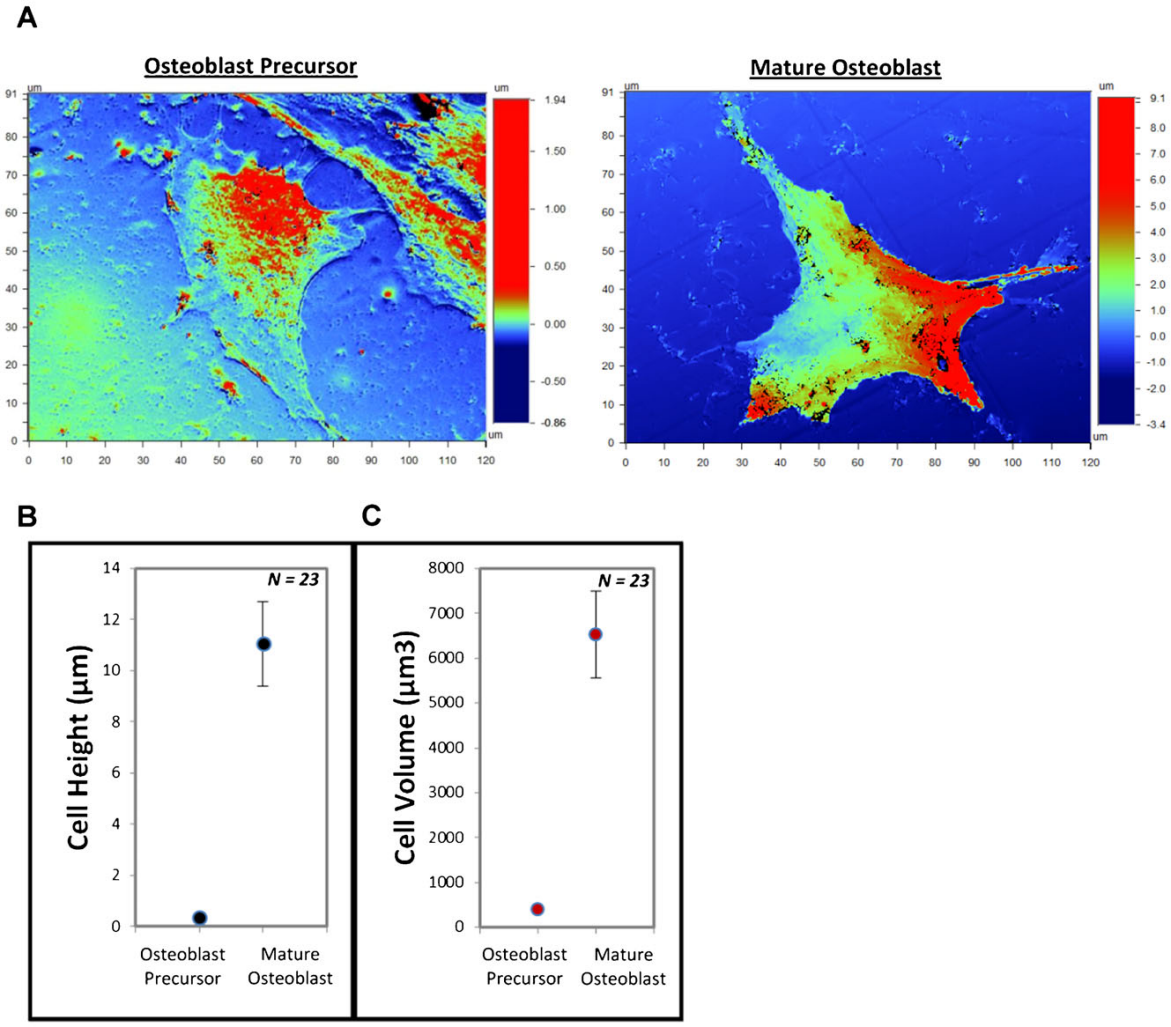
A mature osteoblast characterized by a substantial increase in cell height and volume. (**A**) OPS micrographs showing a single cell on different polymer surfaces after culturing for 24 h. (**B**) A graph quantifying the cell height registered by the OPS after culturing for 24 h. The cell height varies with the level of osteoblast precursor differentiation. (**C**) Volume changes in a single cultured cell.

Accumulating evidence suggests that cell volume alterations are a component of a wide variety of cellular functions, including epithelial transport, metabolism, hormone release, migration, cell proliferation and cell growth and cell death (al-Habori, 1995; Hoffmann et al., 2009). Conceivably, cell volume regulation may play an essential role in cell growth and proliferation (Hoffmann et al., 2009); however, the regulation of cell volume in the osteoblastic lineage is unclear. We demonstrated the characterization of osteoblast precursor differentiation into mature osteoblasts by cell volume modification (Fig. 2A; supplementary material Fig. S2). The optical profilometry technique demonstrated that the cell height and cell volume increased approximately 26-fold in the osteoblast precursors cultured on the polymer functionalized with the BMP-2_mimetic peptide_ after 24 h (Fig. 2B,C). Surprisingly, osteoblast precursors treated with BMP-2 in “the soluble state” do not induce the differentiation from precursors to mature osteoblasts (supplementary material Fig. S3). The data shown here (Figs 1, 2; supplementary material Figs S1–3) demonstrate that BMP-2 retention by covalent binding to the matrix is essential for the induction of osteoblast precursor differentiation into mature osteoblasts.

### Mature osteoblasts are characterized by the novel organization of the cytoskeleton

The role of the cytoskeleton in cell volume regulation has been studied in various cell types (Pedersen et al., 2001). For example, in Ehrlich ascites tumor cells (EATC), cell shrinkage is associated with an increase in cell swelling and a decrease in F-actin content (Pedersen et al., 1999; Pedersen and Hoffmann, 2002). This observation suggests that the effect of F-actin polymerization/depolymerization regulates the process of volume decrease/increase in cells.

**Fig. 3.**
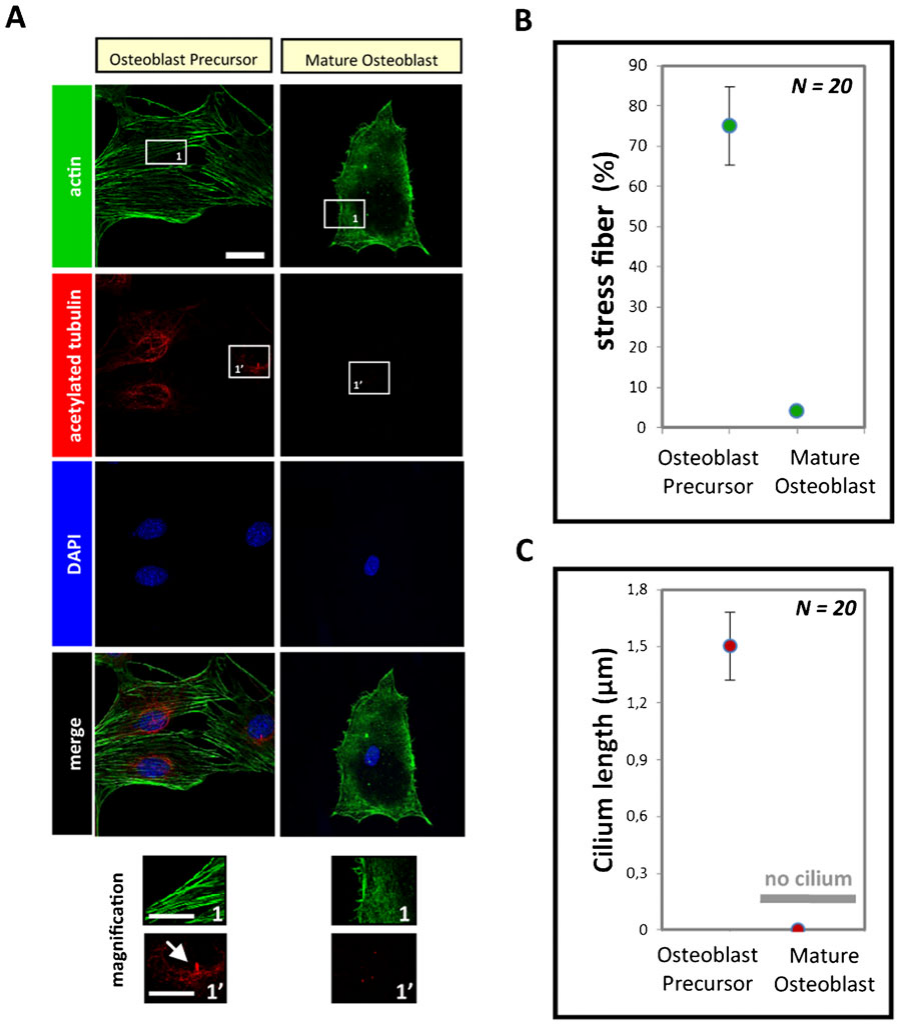
A mature osteoblast characterized by the novel organization of the F-actin cytoskeleton. (**A**) Fluorescence staining of cells under different polymer conditions after culturing for 24 h; the cells were stained for visualization of the actin filaments (green), acetylated tubulin (red) and the nucleus (blue, using DAPI). Scale bars: 10 µm. (**B**,**C**) Graphs quantifying the percentage of stress fibers and cilium length, respectively.

F-actin organization was observed in mature osteoblasts, and a critical decrease in the percentage of stress fiber compared to osteoblast precursors was observed (Fig. 3A,B). This decrease in the F-actin stress fibers correlated with an increase in the cell volume. The shape of the mature osteoblast changed compared to the osteoblast precursor cells (Fig. 3A), and it has been demonstrated that the cell shape and contractility regulate ciliogenesis (Pitaval et al., 2010). To confirm the association between the change in cell shape and dimension with ciliogenesis, we labeled the acetylated tubulin (previously demonstrated to exhibit primary cilia structures) and observed the disappearance of these cytoskeletal structures on the mature osteoblasts (Fig. 3A,C). Short cilia (approximately 1 µm) were observed only on osteoblast precursors (Fig. 3C). Thus, there is evidence that mature osteoblasts play a specific role in bone, and the presence or absence of a number of cytoskeletal structures reflect the commitment of bone cells to a specific function.

### Cell proliferation and nuclear morphology strongly correlate with osteoblast status

The various osteoblastic cell functions interact; for example, previous studies have shown that when cells migrate, the inducting factor (such as BMP-2) induces the cells to migrate and not to differentiate (de Jesus Perez et al., 2009).

**Fig. 4.**
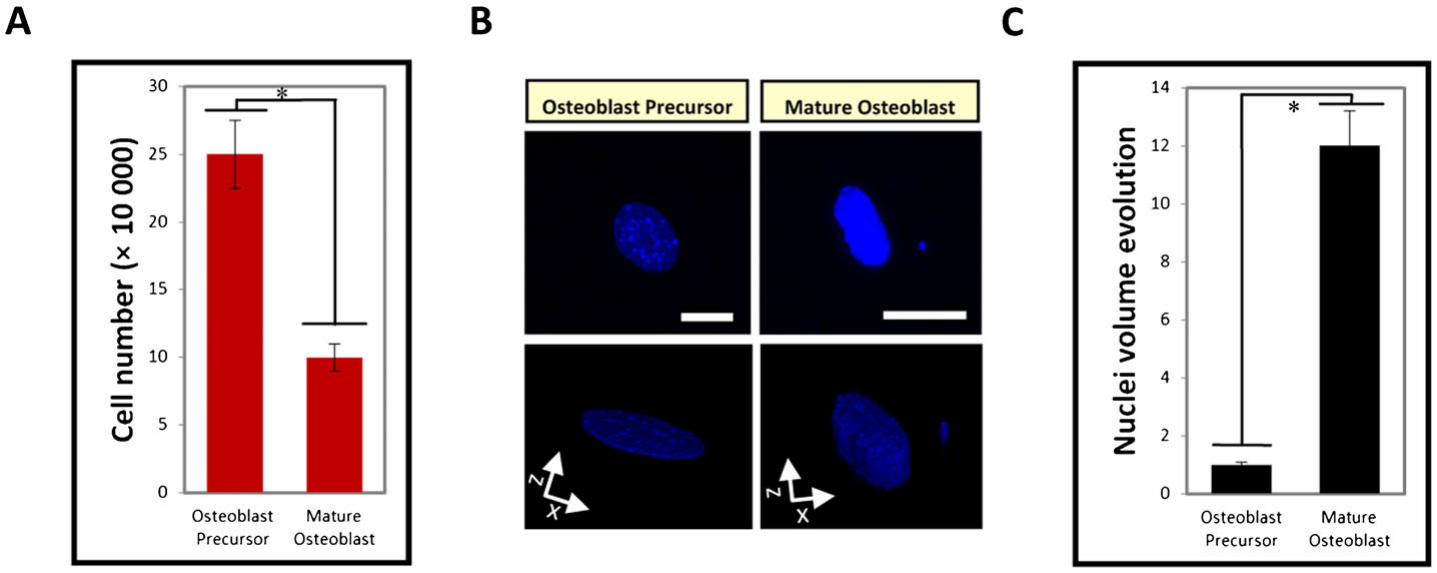
Nuclear morphology strongly correlates with osteoblast status. (**A**) The inhibition of cell proliferation on polymer surfaces promoted the differentiation of osteoblast precursors into mature osteoblasts. (**B**) Top panels: fluorescence staining of the cell nucleus under the two different polymer conditions (native polymer and polymer grafted with the BMP-2_mimetic peptide_) after 24 h. Bottom panels: 3D volumes obtained from the serial confocal sections to confirm that the changes in the diameter represent the changes in the volume. Scale bars: 10 µm. (**C**) A graph quantifying the nuclear volume during differentiation.

The differentiation of osteoblast precursors to mature osteoblasts is characterized by the inhibition of proliferation (Fig. 4A; supplementary material Fig. S4). These results confirm the *in vivo* data suggesting that the function of mature osteoblasts producing ECM to build bone inhibits the cells from performing other functions, such as migration and proliferation. Indeed, precursor osteoblasts, but not mature osteoblasts, move and migrate into developing bones along with invading blood vessels that stabilize the bone (Maes et al., 2010). Interestingly, differentiating osteoblasts are also characterized by changes in their nuclear volume, which increases in mature osteoblasts (Fig. 4B,C). These findings suggest the importance of nuclear morphology on the transformation of osteoblast precursors into mature osteoblasts. These results may also explain the changes in the organization of both the cytoskeleton and the nucleus, which are likely essential for stabilizing the novel cell architecture.

### Runx2 gene expression, integrin engagement and dynamic actin networks regulate osteoblast precursor differentiation into mature osteoblasts

In adherent mature osteoblastic cells, the relationship between the cytoskeleton and the nuclei is suggested by subtle genetic cues; however, this relationship has not been studied extensively and is unclear. Here, we demonstrated that during osteoblast precursors differentiate into mature osteoblasts, the F-actin cytoskeleton is strongly correlated with cell volume (cell height and dimension). Therefore, the next step was to investigate the components that play key roles in this differentiation process and identify the regulators of mature osteoblast functions. The potential of two components was investigated: a cytoplasmic component (F-actin organization) and a genetic component (Runx2 gene expression). In our model, we activated the Smad1/5/8 pathway using BMP-2 mimetic peptides and induced Runx2 gene expression along with other mature osteoblastic markers, such as Col-I. Next, we observed that cell swelling strongly correlated with F-actin cytoskeleton organization and with increased ECM formation. This reasoning suggested that Runx2 gene regulates the reorganization of the actin networks.

**Fig. 5.**
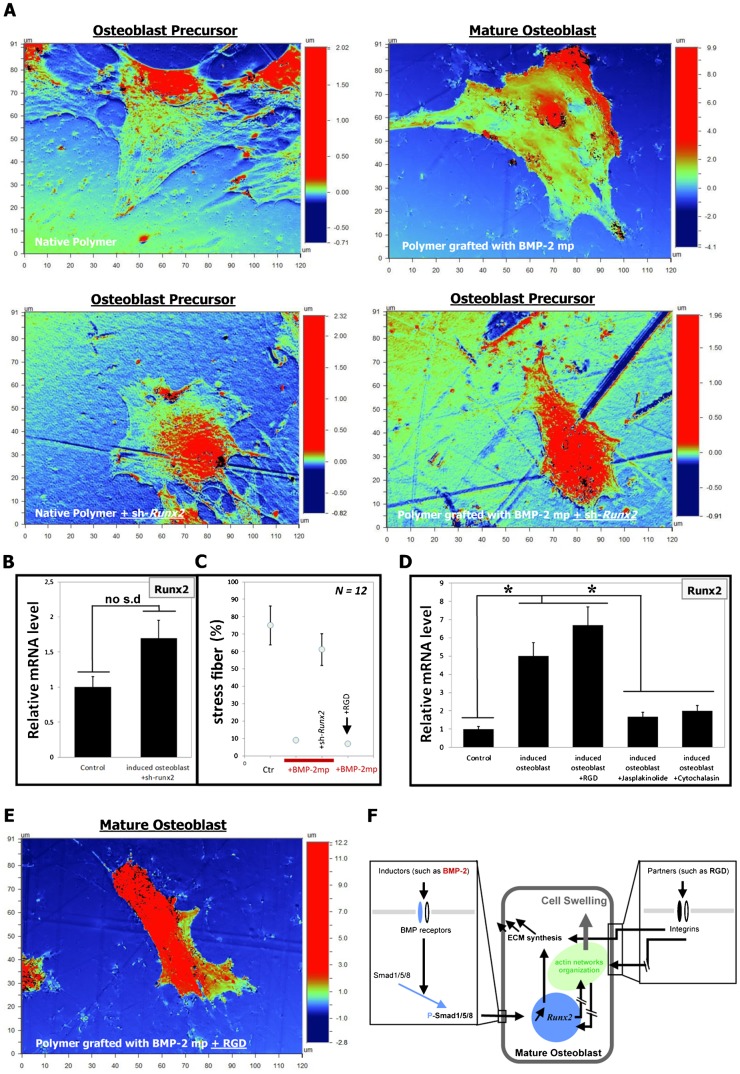
The Runx2 gene regulates osteoblast precursor differentiation into mature osteoblasts via the organization of the actin network. (**A**) OPS micrographs showing that the silencing of Runx2, mediated by shRNA, attenuates the differentiation of osteoblast precursors into mature osteoblasts; therefore, there is no change in the cell height and volume, no increase in Runx2 gene expression (**B**) (no s.d: no significant differences) and no obvious stress fibers (**C**). (**D**) The synchronization of the polymerization of the actin cytoskeleton also attenuates the differentiation of osteoblast precursor cells into mature osteoblasts (*P<0.005). (**E**) OPS micrograph showing the single-cell dimensions of osteoblast precursors cultured for 24 hours on a polymer surface grafted with the BMP-2_mimetic peptides_ and RGD. (**F**) A schematic model illustrating the overall trends of the mechanisms by which Runx2 promotes the differentiation of osteoblast precursors into mature osteoblasts by directing the organization of the actin networks.

To confirm this hypothesis, RNAi was used to silence the Runx2 expression. The silencing of Runx2 gene expression using shRNA inhibited the differentiation of osteoblast precursors into mature osteoblasts (Fig. 5A–C). No significant Runx2 gene over-expression was observed (Fig. 5B); the initial F-actin organization was preserved (stress fiber organization) (Fig. 5C), and there was no cell swelling compared to the induced osteoblasts (Fig. 5A, induced osteoblasts are osteoblast precursors cultured on polymer grafted with BMP-2_mimetic peptide_). These results indicate that Runx2 is required for the differentiation of osteoblast precursors into mature osteoblasts. However, the organization of the F-actin cytoskeleton is also important for the differentiation process. Indeed, jasplakinolide, which stabilizes actin filaments by inhibiting their depolymerization, and cytochalasin D, which inhibits actin polymerization, both inhibit the differentiation of osteoblast precursors into mature osteoblasts (Fig. 5D). The data suggest that the modification of the dynamic of actin networks perturbed the differentiation of the osteoblast precursors into mature osteoblasts.

The addition of adhesion component (RGD peptide) to the differentiation culture model (the polymer grafted with the BMP-2_mimetic peptide_) promotes the differentiation of osteoblast precursors into mature osteoblasts. Indeed, the mature osteoblasts exhibited a higher volume, increased Runx2 gene expression and the highest level of ECM production under these conditions (Fig. 5C,D,E). A decrease in the levels of F-actin stress fibers and the disappearance of acetylated tubulin was also observed (supplementary material Fig. S5). These data suggest that integrin engagement (*via* RGD ligand here) intensifies the differentiation of osteoblast precursors into mature osteoblasts.

## Discussion

The mechanisms that direct the differentiation of osteoblast precursor cells into mature osteoblasts are of interest because of the potential for developing biomaterials that promote bone reparation and regeneration. In this present study, we determined that BMP-2-mediated signaling induces the differentiation of precursor osteoblasts, suggesting the continuous activation of the BMP receptors. Furthermore, we demonstrated the essential role of Runx2 in the differentiation of osteoblast precursors into mature osteoblasts and showed that this differentiation is related to the dynamics of the entire F-actin network.

In this study, we first validated our osteoblast differentiation model by the identification of the following three physiological parameters, which are typical of mature osteoblasts: (i) the significant increase in cell height; (ii) the expression of osteoblastic markers, such as Runx2 and Col-I; and (iii) the production of a large quantity of ECM proteins (Figs 1, 2). Next, we investigated the organization of the F-actin cytoskeleton networks in osteoblast precursors and in mature osteoblasts; mature osteoblasts are characterized by the absence of stress fibers (Fig. 3). It is likely that the reorganization of the F-actin cytoskeleton that promotes the volume changes is accompanied by effects on other cytoskeletal components, such as myosin II, as suggested in other studies (Hoffmann and Pedersen, 2007). However, this may only play a role under extreme conditions of swelling or shear stress. A change in the volume of the cell could certainly initiate different signaling pathways (Pedersen et al., 1999); for example, small G-proteins (the Rho family GTPases Rac1, RhoA and Cdc42, in particular) reversibly associate with the actin cytoskeleton, and this likely plays a role in regulating their activity. Conversely, the Rho GTPases are themselves important regulators of cytoskeletal organization and integrin signaling pathways (Pedersen and Hoffmann, 2002). In our model, when the RGD peptides (derived from natural ECM proteins i.e., fibronectin) and the BMP-2_mimetic peptides_ are grafted onto a polymer surface, the mature osteoblasts exhibit a greater cell volume than the cells cultured on polymers grafted with the BMP-2_mimetic peptide_ alone (Fig. 5). This result supports several studies suggesting that integrin activation also plays a role in sensing the cell volume changes, thus confirming that the engagement of integrins coupled with inductors on a matrix induces the differentiation of osteoblast precursors. The cell swelling during the osteoblast precursor differentiation into mature osteoblasts is associated with a decrease in F-actin stress fiber content (Fig. 3), a process that is observed in many different cell types (Pedersen et al., 1999; Pedersen et al., 2001; Hoffmann and Pedersen, 2007; Hoffmann et al., 2009).

We next investigated whether the differentiation of osteoblast precursors into mature osteoblasts is regulated by Runx2 (genetic regulation) alone or by another cell regulator, such as F-actin. The specific silencing of Runx2 blocked the differentiation of osteoblast precursor cells into mature osteoblasts (Fig. 5). The F-actin networks were synchronized using pharmacological agents during the differentiation of osteoblast precursors on different matrices. This perturbation of the F-actin network dynamic (inhibiting depolymerization/polymerization) blocked the differentiation of osteoblast precursors into mature osteoblasts (Fig. 5). These results suggest that an active actin cytoskeleton dynamic is essential for the generation of mature osteoblasts from precursor osteoblasts.

Mature osteoblasts produced *in vitro* are characterized by novel nuclear dimensions, which also occur in mature osteoblasts *in vivo*, and may be involved in the production of the high levels of ECM proteins. In addition, this observation suggests that there are changes in the elasticity of the nucleus (Hancock and Hadj-Sahraoui, 2009), which likely dictates the F-actin cytoskeleton organization to achieve cell stability. The idea of a mechano-directed pathway between the nucleus and cytoplasm may explain the signaling between Runx2 and the F-actin cytoskeleton organization. A second hypothesis that may explain this relationship is a biochemically directed pathway that utilizes different signaling cascades, targeting proteins that associate with actin, thereby providing a novel insight into the understanding of osteoblast biology.

Our results support the concept that the ECM components regulate BMP signaling (Wang et al., 2008; Hynes, 2009). The binding affinity between the ECM components and BMPs is greater than between the BMPs and their receptors, thus inhibiting the internalization process of the BMPs/BMP receptor complexes, terminating their activity and inducing the correct BMP signaling process. Indeed, Wang et al. demonstrated that type IV collagens regulate BMP-4 signaling in drosophila (Wang et al., 2008). Our study illustrates this concept for the generation of mature osteoblasts.

In summary, we propose that during the differentiation of precursor osteoblasts into mature osteoblasts, changes in osteoblast volume, F-actin cytoskeleton reorganization and Runx2 gene expression occur as shown in Fig. 5F. Furthermore, these results are potentially useful for therapeutics; a number of studies have been published regarding the impact of biomaterials on the behavior of osteoprogenitor cells. For bone repair, the goal has always been to differentiate osteoblasts to produce ECM and induce rapid mineralization (Petite et al., 2000). The overall goal is to generate materials with osteoinductive capacities that are compatible with bone tissue. The majority of published studies promote the use of cell adhesion molecules in the biomaterial; in this case, the osteoblasts proliferate, but the production of the ECM is poor and prolonged. Our study proposes to mimic the ECM and provide a novel tool with which to generate mature osteoblasts, the bone cells responsible for bone formation in mammals. These mature osteoblasts cannot be generated using soluble factors alone (see Materials and Methods for details and supplementary material Fig. S5); therefore, a strategy to generate these cells is required. The results of this study may be exploited to design biomaterials for bone repair.

## Conclusions

It is known that BMPs induce and regulate the fate of cells in the bone. We demonstrated that the differentiation of osteoblast precursors into a mature osteoblasts is induced using the solid BMP-2 induction mode, suggesting that this mode of signaling mimics the BMP-2 signaling that occurs *in vivo* in bone tissue. The differentiation of the osteoblasts is accompanied by dramatic changes in the morphology and dimensions of the cell. The change in the cell dimensions is regulated by the level of Runx2 gene expression, controlling the F-actin cytoskeleton organization. The dynamic of the F-actin networks also controls the differentiation. The finding that BMP signaling is controlled by ECM components and properties provides a novel insight into the understanding of the biology of osteogenesis and for the development and design of biomaterials for engineering bone tissue.

## Materials and Methods

### Polymer surface preparation and the covalent grafting of mimetic peptides

The polymer used in this study is poly(ethylene terephthalate) (PET), a commercial bi-oriented film with a thickness of 100 µm obtained from Goodfellow (France). The surface of this polymer was modified according to the methods of Boxus et al., with some modifications (Boxus et al., 1996; Chollet et al., 2009). The peptide immobilization strategy was performed following a procedure described in previous studies (Chollet et al., 2009; Zouani et al., 2010; Zouani et al., 2013b). Briefly, the materials were treated to create COOH functions on the PET surfaces. Next, the PET-COOH samples were immersed in a solution of dimethylaminopropyl-3-ethylcarbodiimide hydrochloride (EDC, 0.2 M)+N-hydroxysuccinimide (NHS, 0.1 M) in 2-(N-morpholino)-ethanesulfonic acid (MES) buffer (0.1 M in MilliQ water), and the samples were rinsed in MilliQ water (50 mL for 30 min). The same protocol was used for each surface. Finally, the immobilization of the mimetic peptides was achieved using a solution of mimetic peptides/1× PBS (C = 10^−3^ M) incubated for 15 hours at room temperature. After grafting, the disks were rinsed in MilliQ water (100 ml) for 1 week. The following four polymer surfaces were designed: (i) a native polymer, PET; (ii) a polymer grafted with BMP-2_mimetic peptides_ (KIPKACCVPTELSAISMLYL, GENECUST®, FR), PET-BMP-2_mimetic peptides_; (iii) a polymer grafted with cell adhesion peptides (GRGDSPC peptides), PET-GRGDSPC; and (iv) a polymer grafted with cell adhesion and differentiation peptides (GRGDSPC and BMP-2_mimetic peptides_, respectively), PET-GRGDSPC+BMP-2_mimetic peptides_ (C_RGD_ = 10^−3^ M; C_BMP-2 peptides_ = 10^−3^ M). We have indeed performed the same experiments in parallel by grafting a control peptide (KIPKACCVPTEAAAAAMAYA, GENECUST®, FR). We have found significant differences in cell behavior compared to substrates grafted with BMP-2_mimetic peptides_ in terms of adhesion and also differentiation. In fact, no phosphorylation of Smad1/5/8 and no cell swelling have been observed and no ECM has been synthesized by the seeded osteoblast precursor cells (data not shown). The surfaces were characterized using fluorescent peptides and other methods, such as X-ray photoelectron spectroscopy (XPS) and a high-resolution μ-imager. The results of these experiments have been detailed in previous publications (Porté-Durrieu et al., 2004; Chollet et al., 2007a; Chollet et al., 2007b; Chollet et al., 2009; Pallu et al., 2005).

### Cell culture

Mouse pre-osteoblastic cells (MC3T3-E1, from ATCC) were cultured in Alpha-MEM medium supplemented with 10% fetal calf serum (FCS) and 1% penicillin/streptomycin. All cells were used at a low passage number (passage 4), and subconfluent cultures were used; the cells were plated at 10,000 cells/cm^2^ for experiments. For the soluble BMP-2 protein induction mode, the osteoblast precursors were cultured on native polymer substrates and were exposed to 300 ng/mL of recombinant human BMP-2 (Peprotech)-containing media for 24 hours. The synthetic siRNA oligonucleotides specific for regions in the Runx2 mRNA were designed and synthesized by Santa Cruz Biotechnology (sc-37146). The siRNA oligonucleotide was screened for its silencing effect and was initially tested for its ability to knockdown the expression of Runx2 using RT-PCR and immunoblotting. The pharmacological agents used were 1 µM cytochalasin D (Sigma) and 50 nM jasplakinolide (Invitrogen). The osteoblast precursors were exposed to each pharmacological agent for 1 hour, 4 h after seeding on a modified polymer. The cell proliferation was measured using a fluorometric Hoechst 33258 DNA assay.

We cannot compare the results of the osteoblast precursors cultured on the polymers grafted with the BMP-2_mimetic peptide_ and the osteoblast precursors cultured on the native polymer or on plastic treated with soluble BMP-2 protein. First, in our differentiation model, we used a BMP-2_mimetic peptide_ that mimics the monomeric form of the BMP-2 protein. In its soluble state, the BMP-2 protein is mostly homodimeric (supplementary material Fig. S6). Second, there is a difference in the spatial concentration of the BMP-2 in the two systems. In our differentiation model, the osteoblast precursors do not choose their induction intensity because these cells (osteoblast precursors) need to adhere to avoid death. In the BMP-2 soluble mode, the growth factor can remain in the culture media and wait for the availability of a specific BMP receptor. Therefore, we have presented the results of the soluble BMP-2 protein mode in a separate figure (supplementary material Fig. S3).

### Real-time PCR analysis of gene expression

RT-PCR was performed as previously described (Zouani et al., 2010; Zouani et al., 2012). Briefly, total RNA was extracted using the RNeasy total RNA kit from Qiagen in accordance with the manufacturer's instructions. Purified total RNA was used to make cDNA by the reverse transcription reaction (Gibco BRL) using random primers (Invitrogen). Real-time PCR was performed using SYBR green reagents (Bio-Rad). The data were analyzed using iCycler IQ™ software. The cDNA samples (1 µL in a total volume of 20 µL) were analyzed for the gene of interest and for the house-keeping gene HPRT. The comparison test of the cycle-threshold point was used to quantify the gene expression level in each sample. The primers used for the amplification are listed in supplementary material Table S1.

### Western blotting

After 24 hours, cells were permeabilized (10% SDS, 25 mM NaCl, 10 nM pepstatin and 10 nM leupeptin in distilled water and loading buffer), boiled for 10 minutes and resolved by reducing PAGE (Invitrogen). Proteins were transferred onto nitrocellulose, blocked, and labeled with HRP-conjugated antibodies (Invitrogen). Phosphor-Smad1/5/8 was blotted by treating the nitrocellulose with monoclonal anti-p-Smad1/5/8 (Santa Cruz Biotechnology). Our western blot was run in triplicate, along with an additional blot for actin and Coomassie Blue staining to ensure consistent protein load between samples.

### Immunostaining

After 24 hours in culture, the cells on the polymer surfaces were fixed for 20 min in 4% paraformaldehyde/PBS at 4°C. After fixation, the cells were permeabilized in PBS containing 1% Triton X-100 for 15 min. The cytoskeletal filamentous actin (F-actin) was visualized by treating the cells with 5 U/mL Alexa Fluor® 488 phalloidin (Sigma, France) for 1 h at 37°C. The acetylated tubulin was visualized by treating the cells with 1% (v/v) monoclonal anti-acetylated tubulin (produced in mice, Santa Cruz Biotechnology) for 1 hour at 37°C. The *F*(ab)_2_ fragment of rabbit anti-mouse IgG(H+L) was coupled with Alexa Fluor® 568 for 30 min at room temperature. The cell nuclei were counterstained in 20 ng/mL DAPI for 10 min at room temperature. The images for this experiment were produced using a Leica SP5 confocal microscope and MetaMorph software. The Z-series scans were obtained to visualize the staining at different depths. The free Edit 3D software was used for the 3D reconstruction of the confocal images and the nuclear volume calculations. For the quantification of the percentage of stress fibers (F-actin), we used the Image J freeware (Image J, U.S. National Institute of Health, Bethesda, Maryland, USA, 1997–2007, W.S. Rasband, http://rsb.info.nih.gov). After smoothing, the resulting image, which is similar to the original photomicrograph but with minimal background, was converted to a binary image by setting a threshold. The threshold values were determined empirically by selecting a setting (Chollet et al., 2009).

### Histological analysis

For histologic analyses (Monfoulet et al., 2011), undecalcified bones were fixed, dehydrated in increasing ethanol solutions at 4°C, and embedded in methyl methacrylate according to standard protocols. For each right femur, 7-µm-thick longitudinal sections, parallel to the sagittal plane, were obtained using a Leica Polycut E microtome (Leica, Glattburg, Switzerland) equipped with tungsten carbide 50j knives. Sections were stained with aniline blue for osteoblastic cell analysis.

### Scanning electron microscopy (SEM)

Initially, the cells were seeded on the substrates at a density of 1×10^4^ cells/mL. After 24 h in culture under different polymer conditions, the cells were washed with 1× PBS and were fixed with paraformaldehyde in PBS (4%) for 20 min at 4°C. The samples were dehydrated in increasing concentrations of ethanol (30, 70, 80, 90, 95 and 100%) and critical-point dried. The replicas were gold coated and were examined using a scanning electron microscope (SEM Hitachi S2500) at 10 kV.

### Optical profilometry

The Wyko surface profiler systems (Veeco-NT1100) are non-contact optical profilers that use two technologies to measure a wide range of surface heights. The phase-shifting interferometry (PSI) mode allows for the measurement of smooth surfaces and small steps, whereas the vertical scanning interferometry (VSI) mode allows for the measurement of rough surfaces and steps up to several micrometers high. We used the VSI mode to measure the thickness of the extracellular matrix produced by the cells. To compare the surface of PET or PET functionalized with the formed extracellular matrix, we used a spatula to scratch the surfaces. The PSI and VSI modes were used to measure the evolution of cell thickness (cell topography) as follows: the cells were fixed with paraformaldehyde in PBS (4%) for 30 minutes at 4°C and dehydrated with ethanol, and the samples were metallized with gold or titanium. For the detailed calculation of the cell height and cell volume, see supplementary material Fig. S7.

### Cell volume calculation

The cell imaging with OPS involves raster scanning as follows: the profilometer image consists of a three-dimensional map of the apical cell surface with a limited number of points, n, where each raster scan point represents a cell height. Before assessing the cell volume using the OPS, we measured the vertical position (Z*_REF_*) of the probe tip on the substrate where the cells were grown (supplementary material Fig. S7). This process allows us to calculate the real cell height Z(*x*,*y*) by subtracting Z*_REF_* from the measured height value (Z(*x*,*y*)). The cell volume (V_Cell_) can be estimated using the equation:
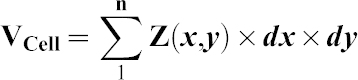
where n is a number of scan points per cell, Z(*x*,*y*) is the cell height at each raster scan point, and *dx* and *dy* are scan increments (steps of 1 µm) in the *x* and *y* directions.

### Statistical analysis

In terms of cell thickness, cell volume, fluorescence intensity, and real-time PCR assay, the data were expressed as the mean ± standard error, and were analyzed using the paired Student's *t*-test method. Significant differences were designated for *P* values of at least <0.01.

## Supplementary Material

Supplementary Material

## References

[b1] al-Habori M. (1995). Microcompartmentation, metabolic channelling and carbohydrate metabolism. Int. J. Biochem. Cell Biol. 27, 123–132 10.1016/1357-2725(94)00079-Q 7767780

[b2] Bonewald L. F. (2011). The amazing osteocyte. J. Bone Miner. Res. 26, 229–238 10.1002/jbmr.320 21254230 PMC3179345

[b3] Boxus T. Deldime-Rubbens M. Mougenot P. Schneider Y.-J. Marchand-Brynaert J. (1996). Chemical assays of end-groups displayed on the surface of poly(ethylene terephthalate) (PET) films and membranes by radiolabeling. Polym. Adv. Technol. 7, 589–598 10.1002/(SICI)1099-1581(199607)7:7<589::AID-PAT551>3.0.CO;2-B

[b4] Carragee E. J. Hurwitz E. L. Weiner B. K. (2011). A critical review of recombinant human bone morphogenetic protein-2 trials in spinal surgery: emerging safety concerns and lessons learned. Spine J. 11, 471–491 10.1016/j.spinee.2011.04.023 21729796

[b5] Chen D. Zhao M. Mundy G. R. (2004). Bone morphogenetic proteins. Growth Factors 22, 233–241 10.1080/08977190412331279890 15621726

[b6] Chen C. H. Tsai F. C. Wang C. C. Lee C. H. (2009). Three-dimensional characterization of active membrane waves on living cells. Phys. Rev. Lett. 103, 238101 10.1103/PhysRevLett.103.238101 20366177

[b7] Chen T. T. Luque A. Lee S. Anderson S. M. Segura T. Iruela-Arispe M. L. (2010). Anchorage of VEGF to the extracellular matrix conveys differential signaling responses to endothelial cells. J. Cell Biol. 188, 595–609 10.1083/jcb.200906044 20176926 PMC2828913

[b8] Chollet C. Chanseau C. Brouillaud B. Durrieu M. C. (2007a). RGD peptides grafting onto poly(ethylene terephthalate) with well controlled densities. Biomol. Eng. 24, 477–482 10.1016/j.bioeng.2007.07.012 17869172

[b9] Chollet C. Labrugère C. Durrieu M. C. (2007b). Impact of RGD peptide density grafted onto Poly(ethylene terephthalate) on MC3T3 cell attachment. Conf. Proc. IEEE Eng. Med. Biol. Soc. 2007, 5123–5126 10.1109/IEMBS.2007.4353493 18003159

[b10] Chollet C. Chanseau C. Remy M. Guignandon A. Bareille R. Labrugère C. Bordenave L. Durrieu M. C. (2009). The effect of RGD density on osteoblast and endothelial cell behavior on RGD-grafted polyethylene terephthalate surfaces. Biomaterials 30, 711–720 10.1016/j.biomaterials.2008.10.033 19010529

[b11] Crouzier T. Fourel L. Boudou T. Albigès-Rizo C. Picart C. (2011). Presentation of BMP-2 from a soft biopolymeric film unveils its activity on cell adhesion and migration. Adv. Mater. 23, H111–H118 10.1002/adma.201004637 21433098

[b12] Davis M. E. Hsieh P. C. Takahashi T. Song Q. Zhang S. Kamm R. D. Grodzinsky A. J. Anversa P. Lee R. T. (2006). Local myocardial insulin-like growth factor 1 (IGF-1) delivery with biotinylated peptide nanofibers improves cell therapy for myocardial infarction. Proc. Natl. Acad. Sci. USA 103, 8155–8160 10.1073/pnas.0602877103 16698918 PMC1472445

[b13] de Jesus Perez V. A. Alastalo T. P. Wu J. C. Axelrod J. D. Cooke J. P. Amieva M. Rabinovitch M. (2009). Bone morphogenetic protein 2 induces pulmonary angiogenesis via Wnt-beta-catenin and Wnt-RhoA-Rac1 pathways. J. Cell Biol. 184, 83–99 10.1083/jcb.200806049 19139264 PMC2615088

[b14] Fan V. H. Tamama K. Au A. Littrell R. Richardson L. B. Wright J. W. Wells A. Griffith L. G. (2007). Tethered epidermal growth factor provides a survival advantage to mesenchymal stem cells. Stem Cells 25, 1241–1251 10.1634/stemcells.2006-0320 17234993

[b15] Gohel A. R. Hand A. R. Gronowicz G. A. (1995). Immunogold localization of beta 1-integrin in bone: effect of glucocorticoids and insulin-like growth factor I on integrins and osteocyte formation. J. Histochem. Cytochem. 43, 1085–1096 10.1177/43.11.7560891 7560891

[b16] Hancock R. Hadj-Sahraoui Y. (2009). Isolation of cell nuclei using inert macromolecules to mimic the crowded cytoplasm. PLoS ONE 4, e7560 10.1371/journal.pone.0007560 19851505 PMC2762040

[b17] Hoffmann E. K. Pedersen S. F. (2007). Shrinkage insensitivity of NKCC1 in myosin II-depleted cytoplasts from Ehrlich ascites tumor cells. Am. J. Physiol. 292, C1854–C1866 10.1152/ajpcell.00474.2006 17229812

[b18] Hoffmann E. K. Lambert I. H. Pedersen S. F. (2009). Physiology of cell volume regulation in vertebrates. Physiol. Rev. 89, 193–277 10.1152/physrev.00037.2007 19126758

[b19] Hynes R. O. (2009). The extracellular matrix: not just pretty fibrils. Science 326, 1216–1219 10.1126/science.1176009 19965464 PMC3536535

[b20] Kato Y. Boskey A. Spevak L. Dallas M. Hori M. Bonewald L. F. (2001). Establishment of an osteoid preosteocyte-like cell MLO-A5 that spontaneously mineralizes in culture. J. Bone Miner. Res. 16, 1622–1633 10.1359/jbmr.2001.16.9.1622 11547831

[b21] Komori T. (2010). Regulation of bone development and extracellular matrix protein genes by RUNX2. Cell Tissue Res. 339, 189–195 10.1007/s00441-009-0832-8 19649655

[b22] Kuhl P. R. Griffith-Cima L. G. (1996). Tethered epidermal growth factor as a paradigm for growth factor-induced stimulation from the solid phase. Nat. Med. 2, 1022–1027 10.1038/nm0996-1022 8782461

[b24] Lei Y. Zouani O. F. Rami L. Chanseau C. Durrieu M. C. (2013). Modulation of lumen formation by microgeometrical bioactive cues and migration mode of actin machinery. Small 9, 1086–1095 10.1002/smll.201202410 23161822

[b100] Lutolf M. P. Blau H. M. (2009). Artificial stem cell niches. Adv. Mater. 21, 3255–3268 10.1002/adma.200802582 20882496 PMC3099745

[b25] Maciel T. T. Kempf H. Campos A. H. (2010). Targeting bone morphogenetic protein signaling on renal and vascular diseases. Curr. Opin. Nephrol. Hypertens. 19, 26–31 10.1097/MNH.0b013e328332fc13 19823085

[b26] Maes C. Kobayashi T. Selig M. K. Torrekens S. Roth S. I. Mackem S. Carmeliet G. Kronenberg H. M. (2010). Osteoblast precursors, but not mature osteoblasts, move into developing and fractured bones along with invading blood vessels. Dev. Cell 19, 329–344 10.1016/j.devcel.2010.07.010 20708594 PMC3540406

[b27] Marie P. J. (2009). Bone cell-matrix protein interactions. Osteoporos. Int. 20, 1037–1042 10.1007/s00198-009-0856-7 19340509

[b28] Mazzaferro S. Pasquali M. Pirrò G. Rotondi S. Tartaglione L. (2010). The bone and the kidney. Arch. Biochem. Biophys. 503, 95–102 10.1016/j.abb.2010.06.028 20599669

[b29] Monfoulet L. E. Rabier B. Dacquin R. Anginot A. Photsavang J. Jurdic P. Vico L. Malaval L. Chassande O. (2011). Thyroid hormone receptor β mediates thyroid hormone effects on bone remodeling and bone mass. J. Bone Miner. Res. 26, 2036–2044 10.1002/jbmr.432 21594896

[b30] Pallu S. Bourget C. Bareille R. Labrugère C. Dard M. Sewing A. Jonczyk A. Vernizeau M. Christine Durrieu M. Amédée-Vilamitjana J. (2005). The effect of cyclo-DfKRG peptide immobilization on titanium on the adhesion and differentiation of human osteoprogenitor cells. Biomaterials 26, 6932–6940 10.1016/j.biomaterials.2005.04.054 15950276

[b31] Pan S. H. Chao Y. C. Hung P. F. Chen H. Y. Yang S. C. Chang Y. L. Wu C. T. Chang C. C. Wang W. L. Chan W. K. (2011). The ability of LCRMP-1 to promote cancer invasion by enhancing filopodia formation is antagonized by CRMP-1. J. Clin. Invest. 121, 3189–3205 10.1172/JCI42975 21747164 PMC3148721

[b32] Pedersen S. F. Hoffmann E. K. (2002). Possible interrelationship between changes in F-actin and myosin II, protein phosphorylation, and cell volume regulation in Ehrlich ascites tumor cells. Exp. Cell Res. 277, 57–73 10.1006/excr.2002.5529 12061817

[b33] Pedersen S. F. Mills J. W. Hoffmann E. K. (1999). Role of the F-actin cytoskeleton in the RVD and RVI processes in Ehrlich ascites tumor cells. Exp. Cell Res. 252, 63–74 10.1006/excr.1999.4615 10502400

[b34] Pedersen S. F. Hoffmann E. K. Mills J. W. (2001). The cytoskeleton and cell volume regulation. Comp. Biochem. Physiol. 130A, 385–399 10.1016/S1095-6433(01)00429-9 11913452

[b35] Petite H. Viateau V. Bensaïd W. Meunier A. de Pollak C. Bourguignon M. Oudina K. Sedel L. Guillemin G. (2000). Tissue-engineered bone regeneration. Nat. Biotechnol. 18, 959–963 10.1038/79449 10973216

[b36] Pitaval A. Tseng Q. Bornens M. Théry M. (2010). Cell shape and contractility regulate ciliogenesis in cell cycle-arrested cells. J. Cell Biol. 191, 303–312 10.1083/jcb.201004003 20956379 PMC2958475

[b37] Porté-Durrieu M. C. Guillemot F. Pallu S. Labrugère C. Brouillaud B. Bareille R. Amédée J. Barthe N. Dard M. Baquey C. (2004). Cyclo-(DfKRG) peptide grafting onto Ti-6Al-4V: physical characterization and interest towards human osteoprogenitor cells adhesion. Biomaterials 25, 4837–4846 10.1016/j.biomaterials.2003.11.037 15120531

[b38] Ramirez F. Rifkin D. B. (2009). Extracellular microfibrils: contextual platforms for TGFbeta and BMP signaling. Curr. Opin. Cell Biol. 21, 616–622 10.1016/j.ceb.2009.05.005 19525102 PMC2767232

[b39] Schmidt J. A. Black J. (1992). Determination of three-dimensional morphometry of adherent cells by surface profilometry. Biomaterials 13, 483–487 10.1016/0142-9612(92)90171-J 1633223

[b40] Sengle G. Charbonneau N. L. Ono R. N. Sasaki T. Alvarez J. Keene D. R. Bächinger H. P. Sakai L. Y. (2008). Targeting of bone morphogenetic protein growth factor complexes to fibrillin. J. Biol. Chem. 283, 13874–13888 10.1074/jbc.M707820200 18339631 PMC2376219

[b41] Sethi N. Kang Y. (2011). Dysregulation of developmental pathways in bone metastasis. Bone 48, 16–22 10.1016/j.bone.2010.07.005 20630490

[b42] Shah A. K. Lazatin J. Sinha R. K. Lennox T. Hickok N. J. Tuan R. S. (1999). Mechanism of BMP-2 stimulated adhesion of osteoblastic cells to titanium alloy. Biol. Cell 91, 131–142 10.1016/S0248-4900(99)80037-9 10399828

[b43] Sieber C. Kopf J. Hiepen C. Knaus P. (2009). Recent advances in BMP receptor signaling. Cytokine Growth Factor Rev. 20, 343–355 10.1016/j.cytogfr.2009.10.007 19897402

[b44] Wagner D. O. Sieber C. Bhushan R. Börgermann J. H. Graf D. Knaus P. (2010). BMPs: from bone to body morphogenetic proteins. Sci. Signal. 3, mr1 10.1126/scisignal.3107mr1 20124549

[b45] Wang X. Harris R. E. Bayston L. J. Ashe H. L. (2008). Type IV collagens regulate BMP signalling in Drosophila. Nature 455, 72–77 10.1038/nature07214 18701888

[b46] Xiao J.-L. Hsu T.-H. Hsu P.-Y. Yang W.-J. Kuo P.-L. Lee C.-H. (2010). Motion of cancer-cell lamellipodia perturbed by laser light of two wavelengths. Appl. Phys. Lett. 97, 203702 10.1063/1.3517448

[b47] Zouani O. F. Chollet C. Guillotin B. Durrieu M. C. (2010). Differentiation of pre-osteoblast cells on poly(ethylene terephthalate) grafted with RGD and/or BMPs mimetic peptides. Biomaterials 31, 8245–8253 10.1016/j.biomaterials.2010.07.042 20667411

[b48] Zouani O. F. Chanseau C. Brouillaud B. Bareille R. Deliane F. Foulc M. P. Mehdi A. Durrieu M. C. (2012). Altered nanofeature size dictates stem cell differentiation. J. Cell Sci. 125, 1217–1224 10.1242/jcs.093229 22302989

[b49] Zouani O. F. Kalisky J. Ibarboure E. Durrieu M. C. (2013a). Effect of BMP-2 from matrices of different stiffnesses for the modulation of stem cell fate. Biomaterials 34, 2157–2166 10.1016/j.biomaterials.2012.12.007 23290467

[b50] Zouani O. F. Lei Y. Durrieu M. C. (2013b). Pericytes, stem-cell-like cells, but not mesenchymal stem cells are recruited to support microvascular tube stabilization. Small [Epub ahead of print] 10.1002/smll.201300124 23625793

