# Supplementary material for: Insights into the osteoblast precursor differentiation towards mature osteoblasts induced by continuous BMP-2 signaling
Source: Biol Open. 2013 Jul 3;2(9):872–81. doi: 10.1242/bio.20134986 (PMC3773333; doi:10.1242/bio.20134986)
Supplement: Supplementary Material [file supp_2_9_872__index.html]

Insights into the osteoblast precursor differentiation towards mature osteoblasts induced by continuous BMP-2 signaling — Supplementary Material 

# Insights into the osteoblast precursor differentiation towards mature osteoblasts induced by continuous BMP-2 signaling

## 

**Files in this Data Supplement:**

- Supplementary Material - Omar F. Zouani et al. doi: 10.1242/bio.20134986
