# Supplementary material for: Insights into the osteoblast precursor differentiation towards mature osteoblasts induced by continuous BMP-2 signaling
Source: Biol Open. 2013 Jul 3;2(9):872–81. doi: 10.1242/bio.20134986 (PMC3773333; doi:10.1242/bio.20134986)
Supplement: Supplementary Material [file supp_bio.20134986_bio.20134986-s1.pdf]

Biology Open

Supplementary Material

Omar F. Zouani et al. doi: 10.1242/bio.20134986

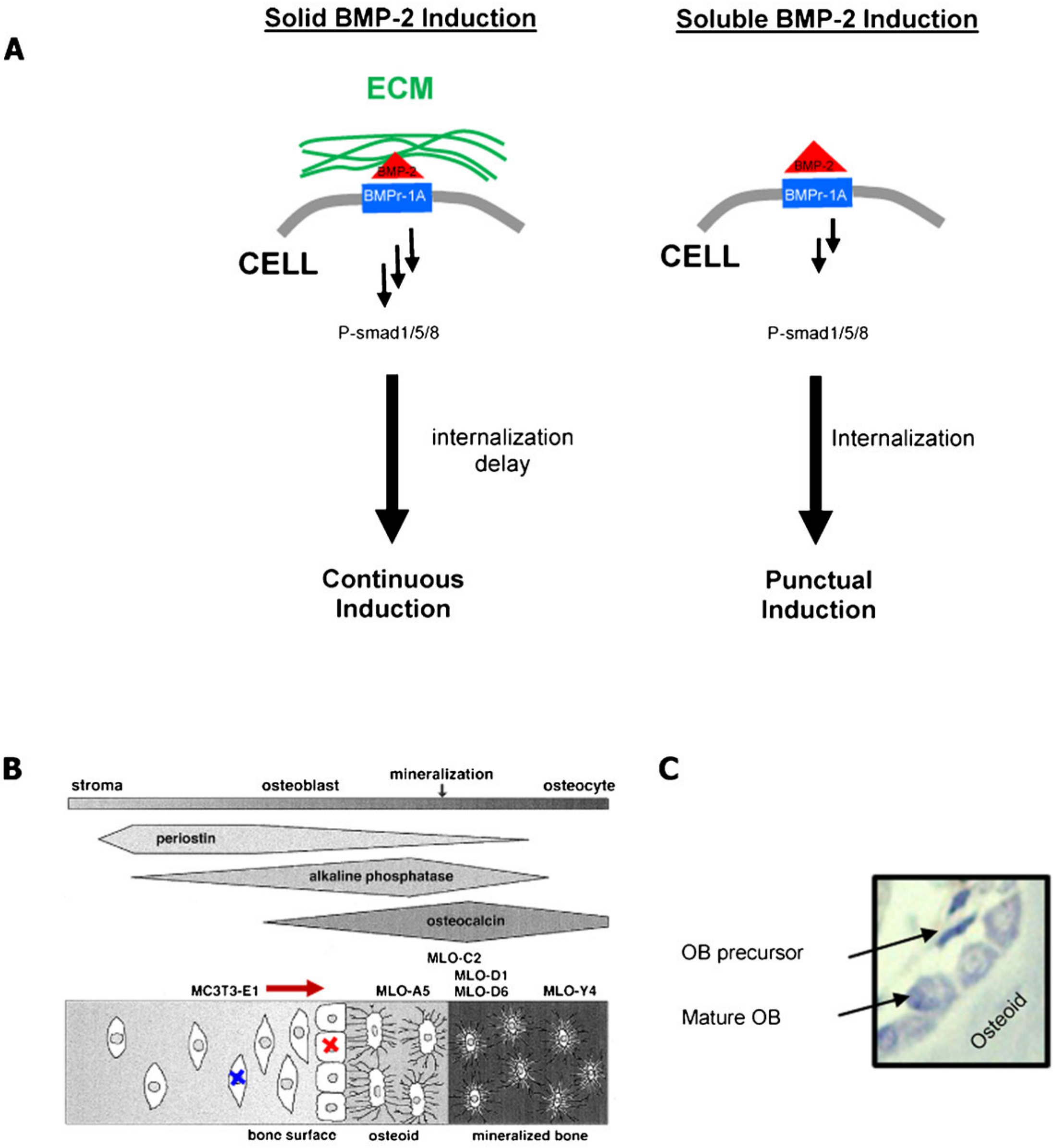

Fig. S1. (**A**) Schematic model illustrated of the overall trends of mechanisms by which BMP-2 induce intracellular signaling pathways. (**B**) Bone cells derived from mesenchymal stem cells and their progression into osteocytes. The diagram depicts various stages of differentiation between osteoblast precursors and mature osteocytes. Osteoblast precursors (blue "+") are required at the bone surface where they differentiate into plump, cuboidal matrix-producing cells, these cells are mature osteoblasts (red "+"). This figure is from Kato et al. (Kato et al., 2001). This scheme is also published with some modification by the same group (Bonewald, 2011). (**C**) Histological analysis of the femur of mice after aniline blue staining (magnification: 40×).

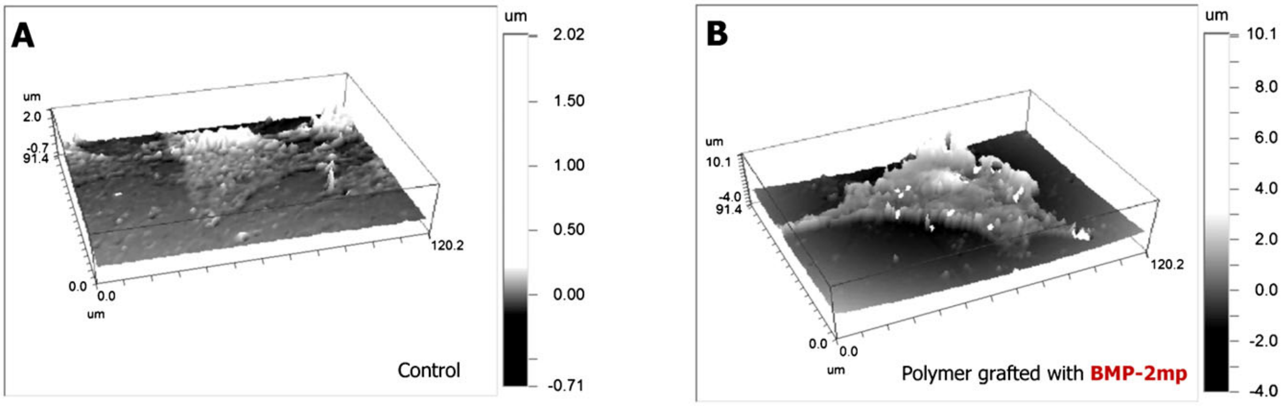

Fig. S2. (**A**,**B**) OPS micrographs (3D reconstruction) showing a single cell on different polymer surfaces after culturing for 24 h.

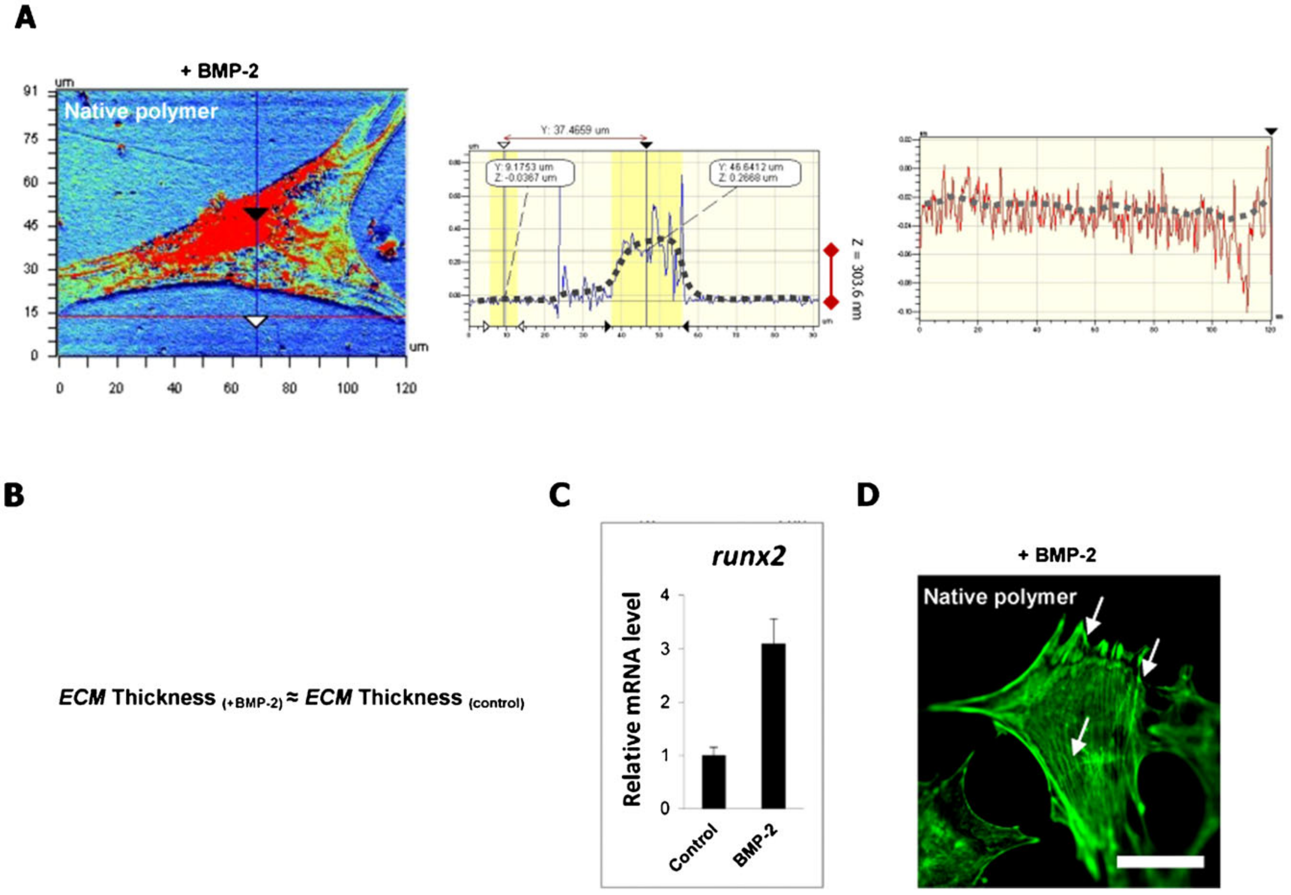

Fig. S3. (**A**) OPS micrograph of osteoblast precursor cultured on native polymer with soluble BMP-2 protein treatment after 24 h. In this condition, cells do not express ECM proteins (**B**), but runx2 gene is expressed (**C**). (**D**) Immunofluorescence image of osteoblast precursors cultured on native polymer with soluble BMP-2 protein treatment showing presence of F-actin stress fiber, here presented in green. Scale bar: 50 μm.

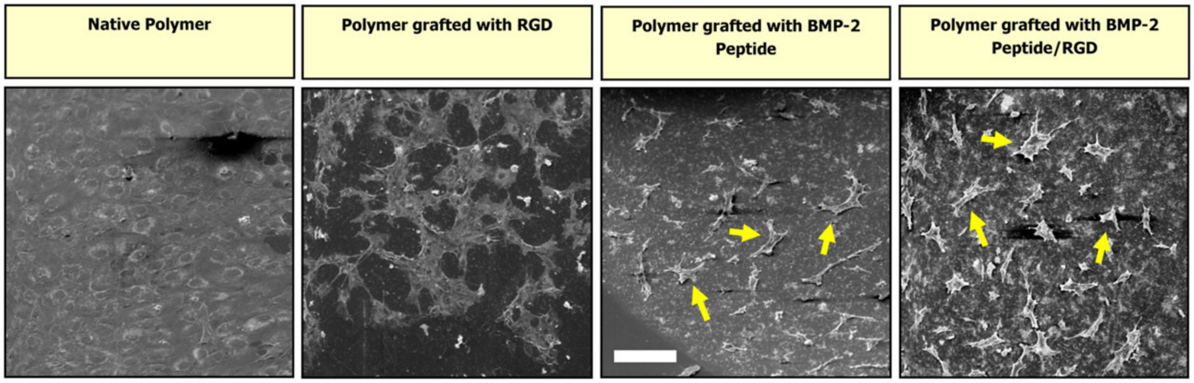

Fig. S4. SEM micrographs of cells cultured for 24 hours on different polymer surfaces. Cell–cell interactions decrease on polymer surfaces when we obtained mature osteoblasts. Scale bar: 100 µm.

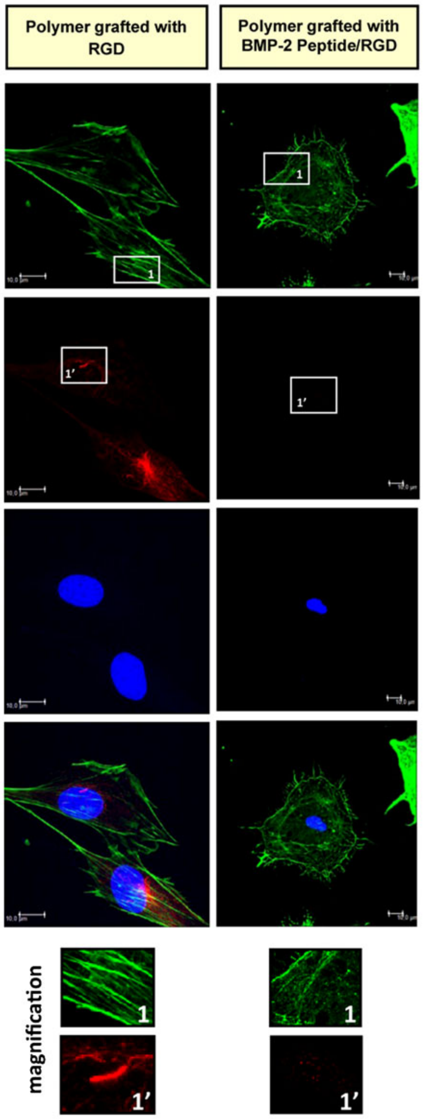

Fig. S5. Fluorescence staining of cells on different polymer conditions (polymer grafted with RGD and polymer grafted with BMP-2 mimetic peptide and RGD) after 24 h of culture, cells were stained for actin filament (green), acetylated tubulin (red) and DAPI for nucleus (blue). Scale bars: 10 µm.

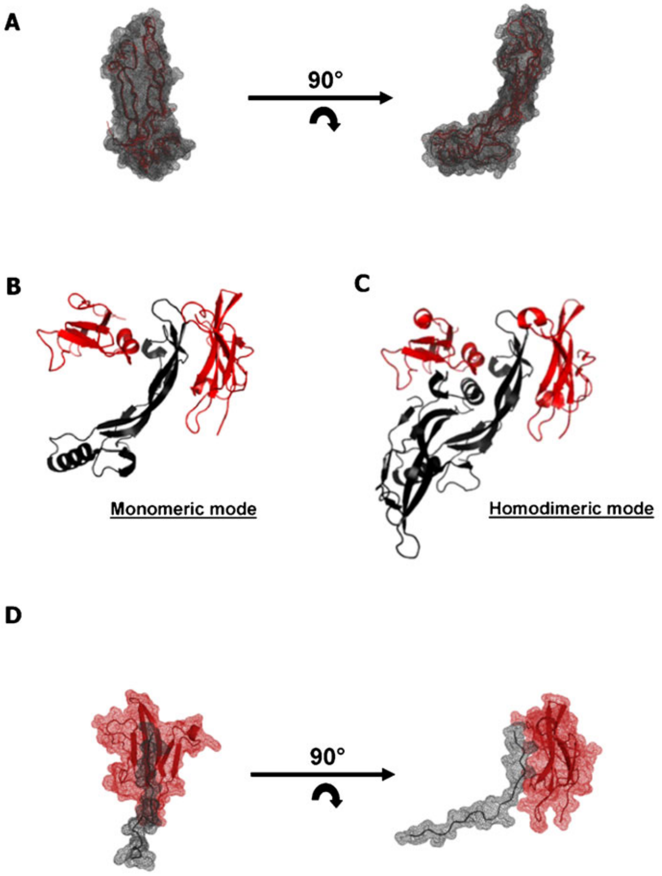

Fig. S6. (**A**) Superposition of BMP family proteins. For clarity only monomers of each protein are displayed: BMP-2, BMP-7 and BMP-9 in red and all members of this family in black. The structure file is downloaded from the PDB. For the BMPs who the structure is unknown, we have predicted their structure with tools cited on SI Materials and Methods. The structures are presented with their electron density map. (**B**) The structure of BMP-$2_{(monomer)}$–BMPr$2_{(ECD)}$ complex. The BMP-2 monomer is represented in black; ECD of BMPr2 is colored in red. (**C**) The structure of BMP-$2_{(homodimer)}$–BMPr$2_{(ECD)}$ complex. The BMP-2 homodimer is represented in black; ECD of BMPr2 is colored in red. (**D**) The predicted structure of the BMP-$2_{mimetic\ peptide}$–BMPr$2_{(ECD)}$. This peptide assures the major interactions with this receptor to activate them.

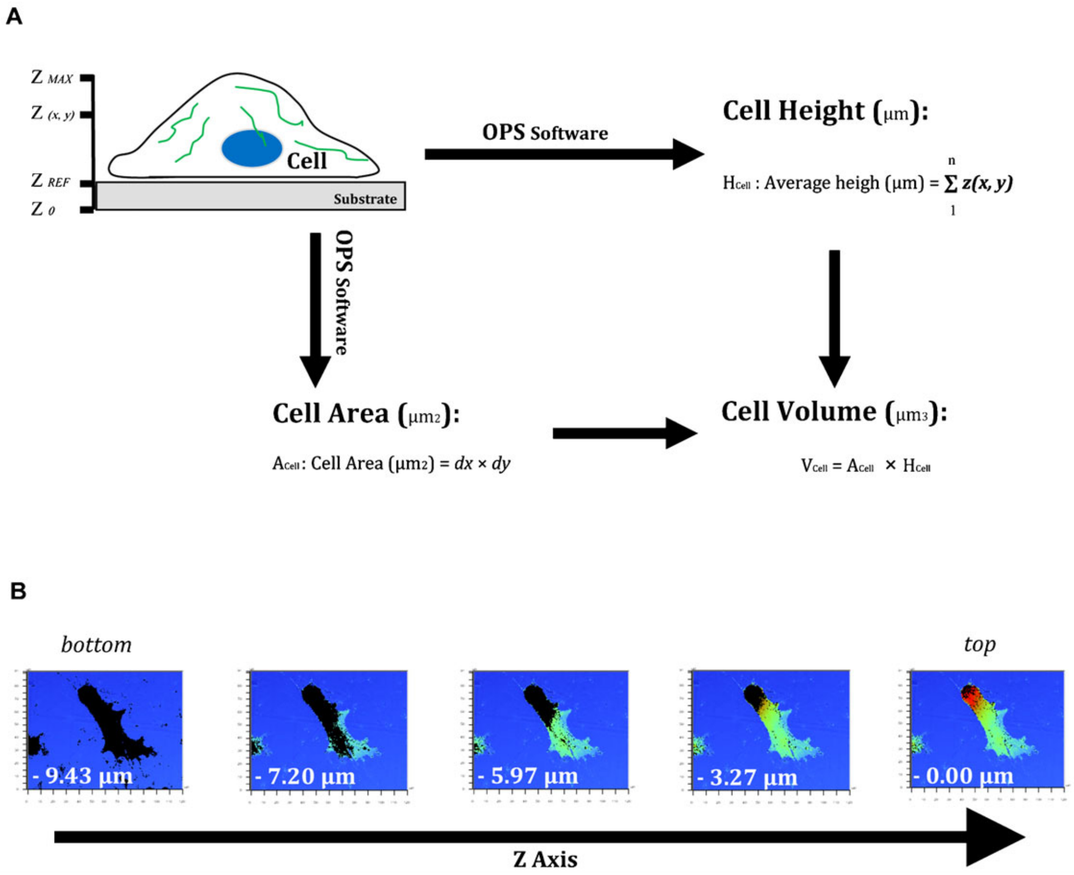

Fig. S7. (**A**) Cell volume calculation. (**B**) OPS micrographs of a mature osteoblast with different z sections.

Table S1. Nucleotide sequences of primers used for quantitative RT-PCR detection.

Gene	Primer sequences
Runx2	5′-GACGTGCCCAGGCGTATTTC-3′ (Forward)<br>5′-AAGTCTGGGGTCCGTCAAGG-3′ (Reverse)
Collagen α1	5′-GAGCGGAGAGTACTGGATCG-3′ (Forward)<br>5′-GCTTCTTTTCCTTGGGGTTC-3′(Reverse)
HPRT	5′-GCAGTACAGCCCCAAAATGG-3′(Forward)<br>5′-ACAAAGTCCGGCCTGTATCCAA-3′(Reverse)
